# Metabolic and ruck performance effects of a novel, light‐weight, energy‐dense ketogenic bar

**DOI:** 10.1113/EP091029

**Published:** 2023-03-13

**Authors:** Alex Buga, Chris D. Crabtree, Justen T. Stoner, Drew D. Decker, Bradley T. Robinson, Madison L. Kackley, Teryn N. Sapper, Jeffrey D. Buxton, Dominic P. D'Agostino, Tyler S. McClure, Anthony Berardi, Shawn Cline, Trevor Fleck, Jared Krout, Doran Newby, Andrew P. Koutnik, Jeff S. Volek, Philip J. Prins

**Affiliations:** ^1^ Department of Human Sciences The Ohio State University Columbus OH USA; ^2^ Department of Exercise Science Grove City College Grove City PA USA; ^3^ Department of Molecular Pharmacology & Physiology University of South Florida Tampa FL USA; ^4^ Human Health, Resilience, and Performance Institute of Human and Machine Cognition Pensacola FL USA

**Keywords:** carbohydrate, exercise, ketogenic, ruck, supplementation, time‐to‐exhaustion

## Abstract

Rucksack marches (‘rucks’) are strenuous, military‐relevant exercises that may benefit from pre‐event fuelling. The purpose of this investigation was to explore whether acute ingestion of carbohydrate‐ or lipid‐based nutritional bars before rucking can elicit unique advantages that augment exercise performance. Recreationally active and healthy males (*n* = 29) were randomized and counterbalanced to consume 1000 kcal derived from a novel, energy‐dense (percentage energy from carbohydrate/fat/protein: 5/83/12) ketogenic bar (KB), or isocaloric high‐carbohydrate bars (CB; 61/23/16) 3 h before a time‐to‐exhaustion (TTE) ruck. Conditions were separated by a 1‐week washout. The rucksack weight was standardized to 30% of bodyweight. Steady‐state treadmill pace was set at 3.2 km/h (0.89 m/s) and 14% grade. TTE was the primary outcome; respiratory exchange ratio (RER), capillary ketones (*R*‐β‐hydroxybutyrate), glucose and lactate, plus subjective thirst/hunger were the secondary outcomes. Mean TTE was similar between conditions (KB: 55 ± 25 vs. CB: 54 ± 22 min; *P =* 0.687). The RER and substrate oxidation rates revealed greater fat and carbohydrate oxidation after the KB and CB, respectively (all *P* < 0.0001). Capillary *R*‐βHB increased modestly after the KB ingestion (*P* < 0.0001). Neither bar influenced glycaemia. Lactate increased during the ruck independent of the condition (*P* < 0.0001). Thirst/fullness perceptions changed independent of the nutritional bar consumed. A novel KB nutritional bar produced equivalent TTE ruck results to the isocaloric CBs. The KB's energy density relative to CB (6.6 vs. 3.8 kcal/g) may provide a lightweight (–42% weight), pre‐event fuelling alternative that does not compromise ruck physical performance.

## Abstract (highlights)

- **What is the central question of the study?**
  Can a novel, energy‐dense and lightweight ketogenic bar (1000 kcal) consumed 3 h before exercise modulate steady‐state incline rucksack march (‘ruck’) performance compared to isocaloric carbohydrate bars in recreationally active, college‐aged men?
- **What is the main finding and its importance?**
  Acute ingestion of either nutritional bar sustained ∼1 h of exhaustive rucking with a 30% of body weight rucksack. This proof‐of‐concept study is the first to demonstrate that carbohydrate bars and lipid bars are equally feasible for preserving ruck performance. Novel ketogenic nutrition bars may have military‐relevant applications to lessen carry load without compromising exercise capacity.

## 1 INTRODUCTION

Soldiers march with heavy rucksacks (rucksack marches; ‘rucks’) in training to simulate long‐range infantry detachment. Rucks are energy intensive physical events that can expend 1000 kcal/h (Walsh et al., 2020), and can contribute significantly to the wide range of total daily energy expenditure observed in active soldiers (3000–7000 kcal/day) (Tassone & Baker, 2017). Energy expenditure monitored during field exercises increases non‐linearly based on bodyweight, carry load, distance, speed and terrain conditions (Army, 2022; Looney et al., 2022; Margolis et al., 2014). Underfeeding for these events is common and is often associated with reduced performance (Jacobs et al., 1989), highlighting the need for operationalizing adequate pre‐event fuelling.

Rations are portable and energy‐dense food items that the military provides to sustain the soldier's nutritional requirements during long patrols or rucks (Hirsch et al., 2005; McClung et al., 2020). However, ration components (main meal + discretionary items) are rarely consumed in their entirety by soldiers, thereby worsening their energy deficits in the field (Hirsch et al., 2005; Huang et al., 2014; Margolis et al., 2014). While exertion and field conditions, specifically environmental temperature, can involuntarily alter appetite and reduce energy intake, voluntary item removal can reduce exposure by up to 30% of total daily calories (approx. 1000–1200 kcal), a detrimental choice for soldiers who are already prone to energetic deficits. (Ahmed et al., 2019).

A logical countermeasure to prevent performance deterioration is to include light and energy‐dense discretionary items that are more widely accepted by soldiers (Hirsch et al., 2005; Huang et al., 2014; Institute of Medicine Committee on Military Nutrition, 1996). Nutrition guidelines stipulate consuming ample carbohydrates (45–65% of total kilocalories) before exercise to elicit ergogenic benefits (American College of Sports et al., 2000; Army, 2022; Defense Logistics Agency, 2022), whereas dietary lipids and proteins have been less studied with respect to performance. In general, criticisms aimed at current field rations stem from containing too many simple carbohydrates (i.e., sugar) and inadequate protein; alternative formulations—with more complex carbohydrates and protein, and less sugar—have recently emerged as a feasible, nutrient‐dense and energy‐adequate option relative to NATO standards for military populations (Lenferna De La Motte et al., 2021). Given the rising interest in alternative nutrition research for the military (Karl et al., 2022; LaFountain et al., 2019), it remains to be determined how acute modifications in macronutrient quality and quantity influences military‐relevant rucking performance.

Several studies have characterized the ∼60–65% maximal aerobic consumption (${\dot{V}}_{O_{2}\max}$) range as the discrete interval where fatty acid (FA) oxidation peaks during submaximal exercise (Achten & Jeukendrup, 2003; Lambert et al., 1997). Rucking is characterized as a moderate‐to‐intense physical activity event (Army, 2022) that may benefit from dietary manipulations to sustain energy supply without the need for refuelling. A plausible mechanism to obviate refuelling is to restrict dietary carbohydrates to a threshold where fat oxidation supersedes carbohydrates for the main substrate utilized during exercise (Helge et al., 2001; Jacobs et al., 1989; Phinney et al., 1983; Volek et al., 2016). The primary expectation is for lipolysis to sustain ATP production, which may concomitantly delay glycogenolysis for intense portions of the ruck (>70% ${\dot{V}}_{O_{2}\max}$), notably if ketones are also present in circulation (Cox et al., 2016; Lambert et al., 1997). While ketogenic diets have been demonstrated as a feasible alternative nutrition strategy to improve body composition and preserve strength and power in military reserve‐officer training corps cadets (LaFountain et al., 2019), it remains to be determined how acute lipid or carbohydrate ingestion in the context of a mixed‐diet influences exercise performance.

Collectively, using the proposed dietary manipulations and recommendations for alternative military nutrition, this study has been designed to explore how acute macronutrient modifications affect military‐relevant rucking performance. To our knowledge, there is no study that has examined the differences between commercially available carbohydrate‐ versus lipid‐dense nutritional bars in a tactically relevant, time‐to‐exhaustion (TTE) ruck trial. This proof‐of‐concept study was devised to compare a TTE ruck with fixed load carrying, speed and grade, done 3 h after ingesting a lipid‐dense ketogenic bar (KB) or isocaloric, carbohydrate‐dense energy bars (CB). Using a randomized and counterbalanced, single‐blind crossover design, the primary objective was to measure TTE and the respiratory and cardiovascular responses during the TTE. Secondary goals were to explore capillary metabolite parameters, such as ketones (*R*‐β‐hydroxybutyrate; *R*‐βHB), glucose, lactate and perceptual measures of thirst and hunger.

## 2 METHODS

### 2.1 Ethical approval

This study was approved by the Institutional Review Board of the Grove City College—Department of Exercise Science (IRB number 124–2020), in accordance with the latest version of the *Declaration of Helsinki* (2013), except for registration in a database. All participants were informed of the experimental procedures, potential risks and the purpose of the study prior to enrolment and before obtaining their written and informed consent.

### 2.2 Participants

This study planned to enrol healthy, recreationally active, college‐aged men. Twenty‐nine males applied and completed all details of the study (Table 1). Participants were included if they (1) currently participated in at least 150 min of moderate activity per week for at least 6 months based on the guidelines by American College of Sports Medicine (Pescatello et al., 2013); (2) were between 18 and 35 years old; and (3) were consuming a Standard American Diet (Shan et al., 2019). Participants were excluded from the study if they (1) had low levels of aerobic fitness (defined as ${\dot{V}}_{O_{2}\max}$ < 40 ml/kg/min); (2) had a history of smoking; (3) had any known metabolic (e.g., diabetes) or cardiovascular disease; (4) had orthopaedic, musculoskeletal, neurological or psychiatric disorders, and/or any medical conditions that prohibit exercise; (5) used any prescription medications; or (6) were following a low‐carbohydrate or ketogenic diet. Participants were prohibited from using any ergogenic aids for 1 month preceding the study and were asked to refrain from taking any performance enhancing supplement(s) during the study. Participants were instructed to refrain from caffeine and alcohol consumption for 48 h and racing or training for 24 h, and food and drink for 8 h before each exercise test. Each participant reported to the laboratory between 06.00 and 09.00 h. Participants were asked to fast for at least 8 h prior to the start of each trial.

**TABLE 1:** Participant characteristics (*n* = 29).

Characteristic	Value
Age (years)	20.4 ± 1.3
Height (cm)	178.1 ± 6.8
Body weight (kg)	77.7 ± 11.5
Body fat (%)	12.6 ± 5.5
Fat free mass (kg)	67.4 ± 7.2
Fat mass (kg)	12.2 ± 11.3
BMI (kg/m^2^)	24.4 ± 2.9
Mean exercise/week (min)	444 ± 216
Exercise experience (years)	7.0 ± 2.8
${\dot{V}}_{O_{2}\max}$ (ml/kg/min)	54.2 ± 7.5

Values are means ± SD. Abbreviations: BMI, body mass index; ${\dot{V}}_{O_{2}\max}$, maximal oxygen consumption.

### 2.3 Study design

**FIGURE 1:**
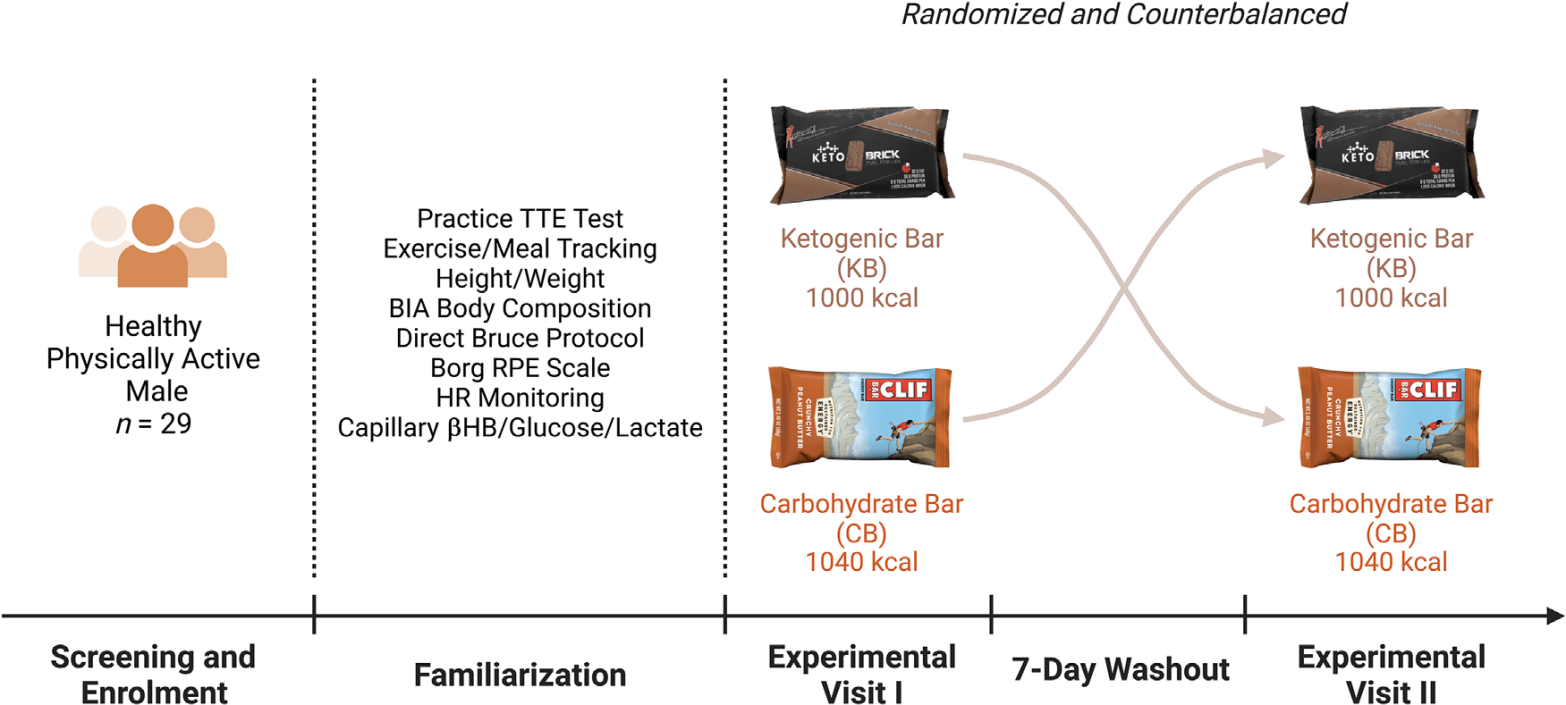
Study design. Healthy, recreationally trained men (*n* = 29) were randomized and counterbalanced to consume 1000 kcal of a ketogenic bar (KB) or isocaloric carbohydrate bars (CB) 3 h before a time‐to‐exhaustion (TTE) treadmill ruck. In‐person sessions were conducted prior to the experimental visits to familiarize the participants with testing procedures and protocols. Experimental visits were separated by at least 7 days to ensure adequate recovery and to minimize carryover effects. BIA, bioelectrical impedance analysis; HR, heart rate; βHB, β‐hydroxybutyrate; RPE, rating of perceived exertion; TTE, time to exhaustion.

A randomized, single‐blind, counterbalanced crossover design was used to assess the effects of consuming either a KB or an isocaloric CB on weighted incline treadmill walking to exhaustion performance. During the first visit, each participant's ${\dot{V}}_{O_{2}\max}$ was determined using a progressive multistage treadmill running protocol (Figure 1).

During subsequent visits, the two main experimental trials consisted of a weighted incline walk TTE test that was completed in a randomized (www.randomizer.org) counterbalanced sequence separated by 7 days. On experimental days, participants consumed either the KB or CB energy bars 3 h prior to performing the TTE test on a treadmill. Capillary glucose, lactate and ketones were measured at baseline, 3 h post energy bar ingestion, at 10 min increments during exercise and immediately following the TTE test. Other variables measured included: (a) TTE, (b) rating of perceived exertion (RPE; RPE‐Overall; RPE‐Chest; RPE‐Legs), (c) heart rate (HR), (d) affect, (e) session RPE, and (f) session affect. RPE, heart rate and affect were taken every 10 min during exercise. In addition, during the TTE test, oxygen consumption (${\dot{V}}_{O_{2}}$), carbon dioxide production (${\dot{V}}_{CO_{2}}$), minute ventilation (${\dot{V}}_{E}$) and respiratory exchange ratio (RER) were assessed and derived from indirect calorimetry. Testing sessions were conducted within the Exercise Science Laboratory of Grove City College at the same time each day at a room temperature between 19 and 21°C and relative humidity of 35–40%.

### 2.4 Pretrial preparation

Participants were instructed to maintain their usual training frequency during the study intervention without increasing or decreasing the training load. The participants were instructed to maintain a training log (mode, duration and intensity of each workout) for 2 weeks before the first experimental trial. They were provided with a copy of their pre‐trial log and instructed to have the same training routine during the intervention period. In addition, participants were asked to record their training every week during the study (mode, duration and intensity of each workout). Furthermore, to quantify the subjects’ training session intensity, participants were asked to record their session RPE (sRPE) after every training session (Lovell et al., 2013) (pre‐trial and within a trial), using the OMNI Walk/Run 0–10 Perceived Exertion Scale (Robertson et al., 2004). The training load for each session was calculated by sRPE × duration of session (minutes) (Foster et al., 2001). The sum of each session's training load provides the quantification of the weekly training load. Training load was assessed each week to measure compliance (Table 2).

**TABLE 2:** Training load.

**Training load**	**Week 1**	**Week 2**	**Week 3**	**Week 4**	**One‐way ANOVA**
RPE × min	2465.7 ± 1536.0	2225.3 ± 1430.0	2018.6 ± 1261.2	2265.5 ± 1187.6	0.107

Data shown as means ± SD.

Participants’ habitual pre‐trial and within‐trial dietary intake was assessed weekly by use of integrative mobile technology (MyFitnessPal, San Francisco, CA, USA). The use of a mobile app for quantifying food intake has been previously shown to be an effective monitoring tool (Turner‐McGrievy et al., 2013). Subjects were asked to keep a detailed diary for 14 days prior to the start of the first experimental trial. Participants were provided with a copy of their pre‐trial log and instructed to have the same dietary intake, specifically during the 2 days before the experimental visit and during the remainder of the study. At the familiarization session participants were given precise oral and written instructions individually on how to accurately record amounts and types of food and beverages. Participants were provided with a digital portable scale (Ozeri ZK 14‐S Pronto, San Diego, CA, USA) and instructed to weigh all food items separately if possible or to estimate the amounts (Table 3).

**TABLE 3:** Nutrient intake.

Variable	Week 1	Week 2	Week 3	Week 4	One‐way ANOVA
Energy (kcal/day)	2340.4 ± 559.4	2411.9 ± 595.6	2325.5 ± 669.1	2391.9 ± 679.4	0.798
Carbohydrate (g)	246.5 ± 92.0	265.0 ± 114.3	234.6 ± 79.4	242.4 ± 109.4	0.211
Protein (g)	125.3 ± 35.7	127.7 ± 46.4	121.3 ± 51.5	128.4 ± 56.6	0.736
Fat (g)	91.6 ± 25.9	97.2 ± 24.6	89.6 ± 26.2	98.3 ± 31.0	0.176
Carbohydrate (%)	41.6 ± 8.2	43.5 ± 12.5	40.9 ± 6.2	40.9 ± 13.6	0.437
Protein (%)	21.8 ± 5.0	21.2 ± 5.8	20.7 ± 5.5	21.1 ± 6.1	0.606
Fat (%)	35.4 ± 8.1	36.9 ± 7.4	35.2 ± 5.6	38.2 ± 9.2	0.227
Cholesterol (mg)	478.3 ± 244.5	451.7 ± 245.8	397.9 ± 196.8	454.9 ± 287.1	0.104
Sodium (mg)	3282.5 ± 1675.9	3406.2 ± 1778.1	3271.9 ± 1382.7	3384.9 ± 2007.9	0.872
Fibre (g)	17.3 ± 5.8	17.2 ± 10.3	17.6 ± 8.4	22.7 ± 33.9	0.559
Sugar (g)	79.7 ± 48.5	82.2 ± 47.5	73.1 ± 38.9	68.2 ± 40.8	0.143

Data shown as means ± SD. Participants (*n* = 29) adhered to a Standard American Diet (Shan et al., 2019) through the intervention timeline.

### 2.5 Familiarization and anthropometric measurements

At the first laboratory visit, all the experimental procedures were explained to the participants. The participants underwent an orientation involving the practice of the TTE test, the various measurement instruments, equipment, affect measures and perceived exertion. Affect was measured using a validated 11‐point Feeling Scale (Hardy, 1989), with participants informed that their responses should reflect the affective or emotional components of the exercise and not the physical sensation of effort or strain. The OMNI Walk/Run Perceived Exertion Scale (Robertson et al., 2004) was used to measure the physical perceptions of exertion for the overall body (RPE‐O), legs (RPE‐L) and chest (RPE‐C). Following the orientation session, anthropometric measures were obtained including height (cm), weight (kg), fat free mass (kg) and fat mass (% and kg). Height (cm) was measured using a physician's scale (Detecto, Webb City, MO, USA). Participants' body mass (kg) and body composition (fat and lean mass) were measured using a Tanita bioelectrical impedance analyser (MC‐980Uplus, Tanita Corporation of America, Arlington Heights, IL, USA). Finally, the participants performed a weighted ruck familiarization trial on a motorized treadmill (Trackmaster TMX425C treadmill, Newton, KS, USA).

### 2.6 Maximal aerobic capacity

On the second laboratory visit, participants performed an incremental test to exhaustion on a motorized treadmill (TMX425C treadmill). ${\dot{V}}_{O_{2}}$ and ${\dot{V}}_{CO_{2}}$ production were measured using an automated metabolic analyser system (TrueOne 2400, ParvoMedics, Sandy, UT, USA) calibrated prior to each exercise test using standard calibration gases (16% O_2_ and 4% CO_2_). Participants wore a Polar heart rate monitor (H10, Polar Electro, Kempele, Finland) during exercise to measure heart rate. The Bruce protocol was used to evaluate ${\dot{V}}_{O_{2}\max}$ (Bruce, 1981). In this protocol, speed and grade of the treadmill increases every 3 min until volitional exhaustion. At least two of the following criteria were required for the maximal exercise test to be considered valid: (1) achievement of maximum HR ≥85% of the age‐predicted maximum; (2) RPE ≥9; (3) respiratory exchange ratio > 1.1 at maximal exertion; or (d) ${\dot{V}}_{O_{2}}$ plateau despite further increase in workload. At the end of the test, the highest average ${\dot{V}}_{O_{2}}$ value recorded over a 30 s period of exercise was considered the participant's ${\dot{V}}_{O_{2}\max}$.

### 2.7 Experimental protocol

**FIGURE 2:**
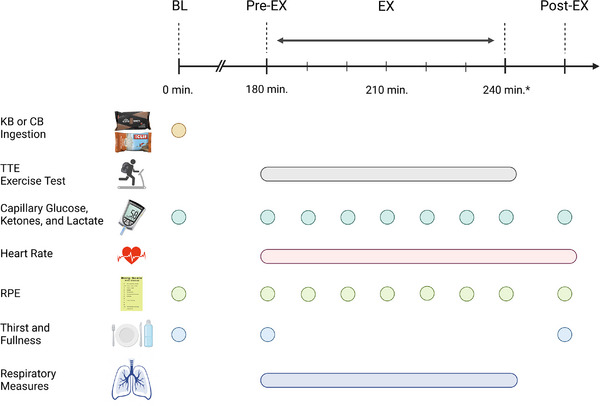
Experimental visit time line. Participants (*n* = 29) completed two (KB or CB) cross‐over experimental visits in random order. Visits stipulated consuming a supplement bar (KB or CB) shortly after lab arrival, while fasted. Three hours post‐ingestion, participants began the ruck time‐to‐exhaustion (TTE) trial until volitional termination. Performance, physiological, metabolic and perceptual measures were collected pre‐, during and post‐exercise. *Average TTE completion time was approximated to 1 h. BL, baseline; CB, carbohydrate bars; EX, exercise; KB, ketogenic bar; min, minutes elapsed; RPE, rate of perceived exertion.

During the experimental visits, participants reported to the lab after an 8 h fast to consume either one serving of the KB (Keto Brick Inc., Bryant, AR, USA; 1000 kcal) or four servings of the CB (Clif Bar Inc., Berkeley, CA, USA; 1040 kcal) with the percentage of total energy distribution from carbohydrate/fat/protein as 5/83/12 and 61/23/16, respectively. More detailed characteristics of each bar are provided in Table 4. Participants were also provided with 500 ml of water to consume ad libitum. Each condition was separated by at least 1 week. The crunchy peanut butter flavour of the CB was used because it most closely matched the KB for calories and contains a contrasting macronutrient composition. Testing was single‐blinded as participants did not know the type of nutrition bar consumed. The blinding was achieved by removing the wrappers from the bars and cutting each bar into bite‐size portions given the energy bars were similar in appearance (i.e., the same colour and consistency) (Figure 2).

**TABLE 4:** Nutrition bar characteristics.

**Description**	**Ketogenic bar (KB)**	**Carbohydrate bars (CB)**	**Net difference (KB − CB)**
Total energy consumed (kcal)	1000	1040	−40
Total weight (g)	151	272	−121
Energy density (kcal/g)	6.6	3.8	2.8
Total volume (ml)	162	236	−74
Physical density (g/ml)	0.93	1.16	−0.23
Fat (g)	92	32	60
Saturated fat (g)	56	4	52
Polyunsaturated fat (g)	6	12	−6
Monounsaturated fat (g)	30	14	16
Medium chain triglycerides (g)	20	0	20
Carbohydrate (g)	12	160	−148
Sugar (g)	0	68	−68
Fibre (g)	8	20	−12
Protein (g)	32	44	−12
Sodium (mg)	750	920	−170
Price per 1000 kcal ($USD)	$12.00	$7.27	$4.73
Price per kilogram ($USD)	$79.47	$27.79	$51.68

CB represents four full servings (4 × 68 g). Total nutritional bar volume and physical density were determined using water displacement (water temperature: 20°C). Medium chain triglycerides provided 70% C8 + 30% C10 bound to acacia fibre. Other nutritional values are reported from the nutrition bar label. Price (United States dollar; $USD) was calculated from the manufacturer's listed market value for single unit purchase of KB ($12.00) and CB ($1.89).

### 2.8 Blood sampling

Fingertip (capillary) blood samples for blood *R‐*βHB (Precision Xtra, Abbott Diabetes Care Inc., Almeda, CA, USA), glucose (Precision Xtra, Abbott Diabetes Care Inc.) and lactate (Lactate Plus, Nova Biomedical, Waltham, MA, USA) concentrations were measured at baseline, 3 h post energy bar ingestion, at 10‐min increments during exercise, and immediately following the TTE test. Samples were collected using a lancet following cleaning of the fingertip with an alcohol swab and then dried. The first droplet was wiped away with a cotton swab to remove any alcohol and the subsequent droplets were used for analysis. Blood sampling during exercise lasted ∼30–60 s – at each 10 min increment of exercise, the participant straddled the treadmill for a 1 min period to allow a capillary blood sample to be taken for subsequent blood *R*‐βHB, glucose and lactate analysis.

### 2.9 Time to exhaustion test

To determine exercise performance, participants performed a weighted ruck TTE test on a motorized treadmill (TMX425C treadmill). Participants were instructed to walk for as long as possible. Participants were provided with feedback on the time (at regular 10 min intervals) covered during each TTE test but were not informed of the overall performance time until the completion of the study. The timing devices were concealed from the participant's view throughout the test. The TTE test consisted of walking on a treadmill wearing a military backpack weighing 30% of the subject's body weight (23.3 ± 3.5 kg), as accepted during operational missions (Army, 2022) in comfortable environmental conditions (19–21°C, 35–40% relative humidity). After a 5 min warm‐up (2 mi/h; 3.2 km/h; 0.89 m/s; 2% slope), the pace remained the same and the slope increased to 14%, until reaching volitional exhaustion. Heart rate (Polar Electro) and metabolic gases were continuously collected during the entire protocol using a metabolic cart for assessment of RER, ${\dot{V}}_{O_{2}}$, ${\dot{V}}_{CO_{2}}$, ${\dot{V}}_{E}$, HR and substrate oxidation.

### 2.10 Perceptual measurements

Participants’ RPE (RPE‐Overall; RPE‐Chest; RPE‐Legs) and affect (Feeling Scale) were recorded at 10 min intervals during the TTE protocol. Ratings of perceived exertion and affect for the entire exercise session (session RPE and session affect) were obtained 5 min following the test. Thirst and gut fullness levels were measured at baseline, 3 h post energy bar ingestion and post‐TTE trial on a scale 1–7 (1: ‘not thirsty at all’, 7: ‘very, very thirsty’) and on a scale 0–10 (0: ‘empty’, 10: ‘extremely full’) (Engell et al., 1987).

### 2.11 Statistical analysis

Statistical analyses were performed using SPSS Statistics version 26.0 (IBM Corp., Armonk, NY, USA). Statistical significance was set a priori at *P* < 0.05. Descriptive statistics were calculated for all variables. Data were tested for normality using the Shapiro–Wilk test. Performance data such as TTE, mean exercise HR, RER, ${\dot{V}}_{O_{2}}$, ${\dot{V}}_{CO_{2}}$, ${\dot{V}}_{E}$, carbohydrate oxidation, fat oxidation, affect, RPE‐Chest, RPE‐Legs, RPE‐Overall, session RPE and session affect were analysed using a paired‐samples Student's *t*‐test as overall means throughout the exercise session. A 2 (condition, KB vs. CB) × 4 (baseline, 3 h post, exercise and immediately post exercise) repeated measures ANOVA was conducted to assess the effect of time, treatment and interaction between time and treatment, on plasma glucose, ketones and lactate. A 2 × 3 (baseline, 3 h post and immediately post‐exercise) repeated measures ANOVA was conducted to assess the effect of time, treatment and interaction between time and treatment on subjective measures of thirst and fullness. A one‐way repeated measures ANOVA was used to analyse differences over time for training load and nutrient intake before and during the intervention. Post‐hoc analyses of significant main and interaction effects were conducted where appropriate using the Bonferroni adjustment to determine which conditions were significantly different. The assumption of sphericity was confirmed using Mauchly's test. Greenhouse–Geisser epsilon corrections were used when the sphericity assumption was violated. Effect sizes (Cohen's *d*) were calculated and interpreted as: small effect > 0.2; medium effect > 0.5; large effect > 0.8.

## 3 RESULTS

### 3.1 Performance

All participants (*n* = 29) were included in the final analysis (Table 5). Self‐reported daily energy intake, macronutrient composition and exercise intensity were maintained between the experimental visits and wash‐out. There were no order effects between visits, evidencing proper counterbalancing, randomization and wash‐out duration.

**TABLE 5:** Time to exhaustion (TTE) results (*n* = 29).

Variable	KB	CB	*P*	Effect size
TTE performance (min)	55.1 ± 24.9	53.9 ± 21.6	0.687	0.05
HR (beats/min)	151.5 ± 18.4	151.3 ± 19.7	0.945	0.01
RPE‐O	5.9 ± 1.2	6.3 ± 1.4	0.063	0.31
RPE‐C	4.9 ± 1.9	5.0 ± 1.9	0.647	0.05
RPE‐L	5.3 ± 1.6	5.4 ± 1.7	0.647	0.06
Affect	−0.5 ± 1.7	−0.7 ± 1.5	0.432	0.12
${\dot{V}}_{O_{2}}$ (l/min)	2.16 ± 0.44	2.17 ± 0.44	0.932	0.02
${\dot{V}}_{O_{2}}$ (ml/kg/min)	28.0 ± 4.5	27.9 ± 4.2	0.925	0.02
Mean ${\dot{V}}_{O_{2}}$ (% of ${\dot{V}}_{O_{2}\max}$)	52.8 ± 12.1	52.3 ± 10.6	0.709	0.04
${\dot{V}}_{CO_{2}}$ (l/min)	1.85 ± 0.39	1.97 ± 0.37	**0.024**	0.32
${\dot{V}}_{E}$ (l/min)	55.5 ± 13.5	57.9 ± 12.9	0.219	0.18
RER	0.85 ± 0.05	0.91 ± 0.06	**<0.0001**	1.09
Carbohydrate oxidation (g/min)	1.47 ± 0.63	2.04 ± 0.65	**<0.0001**	0.87
Fat oxidation (g/min)	0.52 ± 0.23	0.35 ± 0.26	**<0.0001**	0.78
Session RPE	6.9 ± 1.7	7.1 ± 1.4	0.510	0.13
Session affect	−0.8 ± 1.9	−0.9 ± 1.6	0.792	0.06

Data shown as means ± SD. Effect sizes (Cohen's *d*): <0.2: trivial; 0.2–0.49: small; 0.5–0.79: moderate; >0.8: large. *P*‐values shown in bold are statistically significant. CB, carbohydrate bars; HR, heart rate; KB, ketogenic bar; RER, respiratory exchange ratio; RPE, rating of perceived exertion (OMNI rating of exertion); RPE‐C, RPE for chest; RPE‐L, RPE for legs; RPE‐O, RPE for overall body; TTE, time to exhaustion.

When analysing the conditions individually, there were no significant mean differences (∆) between the mean TTE achieved on the KB and CB (∆: 1.2 ± 16.1 min; *P* = 0.687). Secondary performance parameters such as heart rate, fatigue, affect, mean ${\dot{V}}_{O_{2}}$ and session affect were also unaffected by condition.

When combining the results from both the KB and CB conditions, participants terminated the ruck exercise after ∼55 min (range: 21–115 min). Mean work capacity during the TTE was 53% ± 11% of ${\dot{V}}_{O_{2}\max}$.

### 3.2 Metabolic responses

Significant changes were observed in RER and mean substrate oxidation during exercise. The KB condition produced significantly lower mean RER values (∆: −0.06 ± 0.05; *P* < 0.0001) and lower expired mean ${\dot{V}}_{CO_{2}}$ (∆: −0.12 ± 0.27 l/min; *P* = 0.024) compared to the CB. Based on RER there was an 18% increase in fat or carbohydrate oxidation preference during exercise, 3 h after consuming the KB or CB, respectively.

**FIGURE 3:**
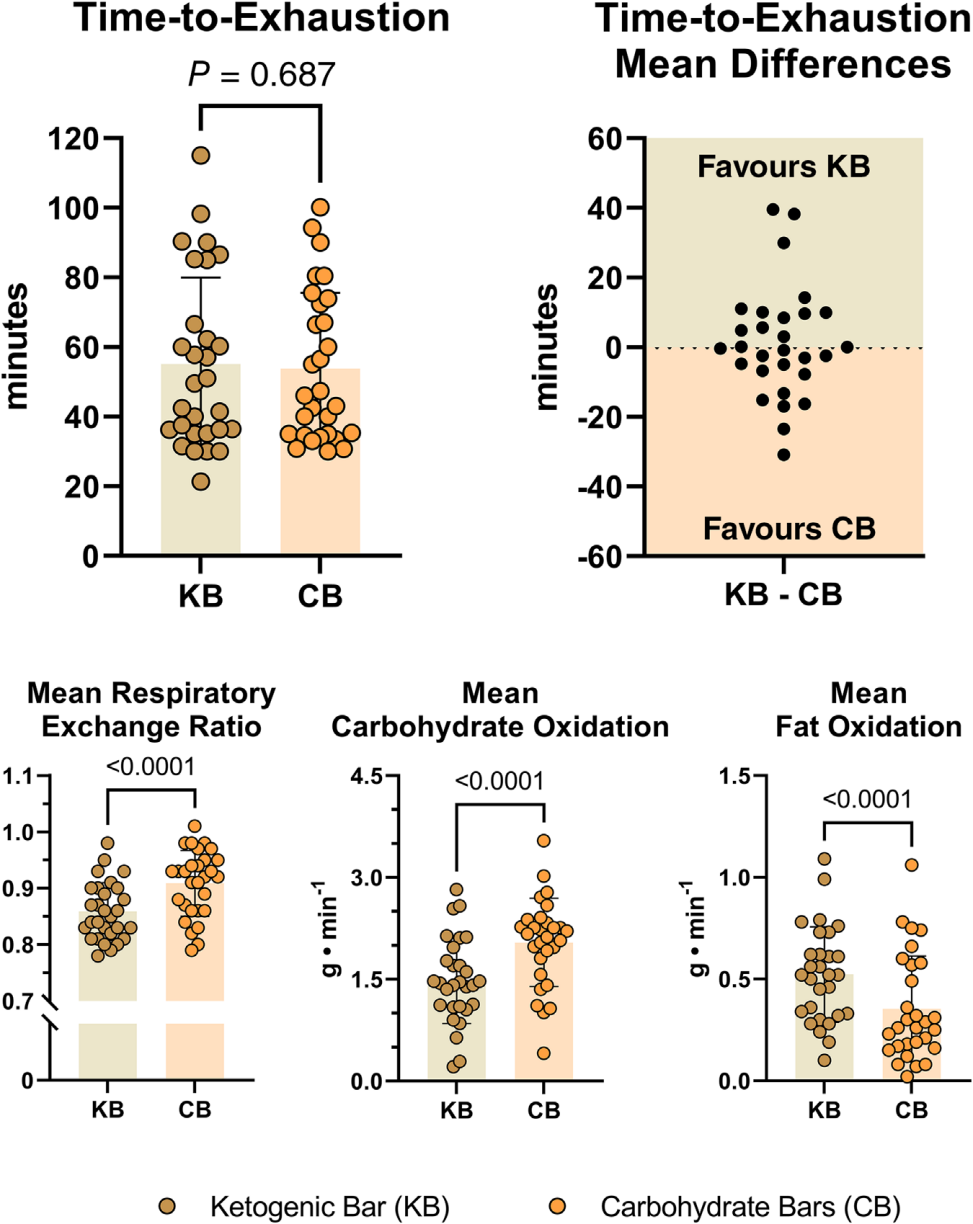
Performance results. All participants (*n* = 29) completed the treadmill‐based ruck time‐to‐exhaustion (TTE) ruck under continuous gas exchange sampling. Dependent *t*‐tests revealed that the KB did not significantly modulate the mean TTE ruck performance beyond isocaloric servings of the CB consumed 3 h before exercise. Mean respiratory exchange ratio and carbohydrate oxidation increased during the CB condition whereas fat oxidation increased during the KB condition, reflecting the macronutrient composition of the bar consumed pre‐exercise (all *P* < 0.0001). Data presented as mean ± SD. CB, carbohydrate bars; KB, ketogenic bar.

Mean fat oxidation rate increased significantly during the KB TTE trial versus CB (∆: 0.17 ± 0.20 g/min; *P* < 0.0001), whereas the CB elicited greater mean carbohydrate oxidation relative to the KB (∆: 0.57 ± 0.61 g/min; *P* < 0.0001). When converting the differences in oxidation rate (g/min) to total kilocalories per hour, the KB condition expended an extra 92 kcal/h from lipids whereas CB expended an extra 137 kcal/h from carbohydrates to sustain equal workload (1:1.5 fat‐to‐carbohydrate ratio) (Figure 3).

### 3.3 Capillary blood parameters

**FIGURE 4:**
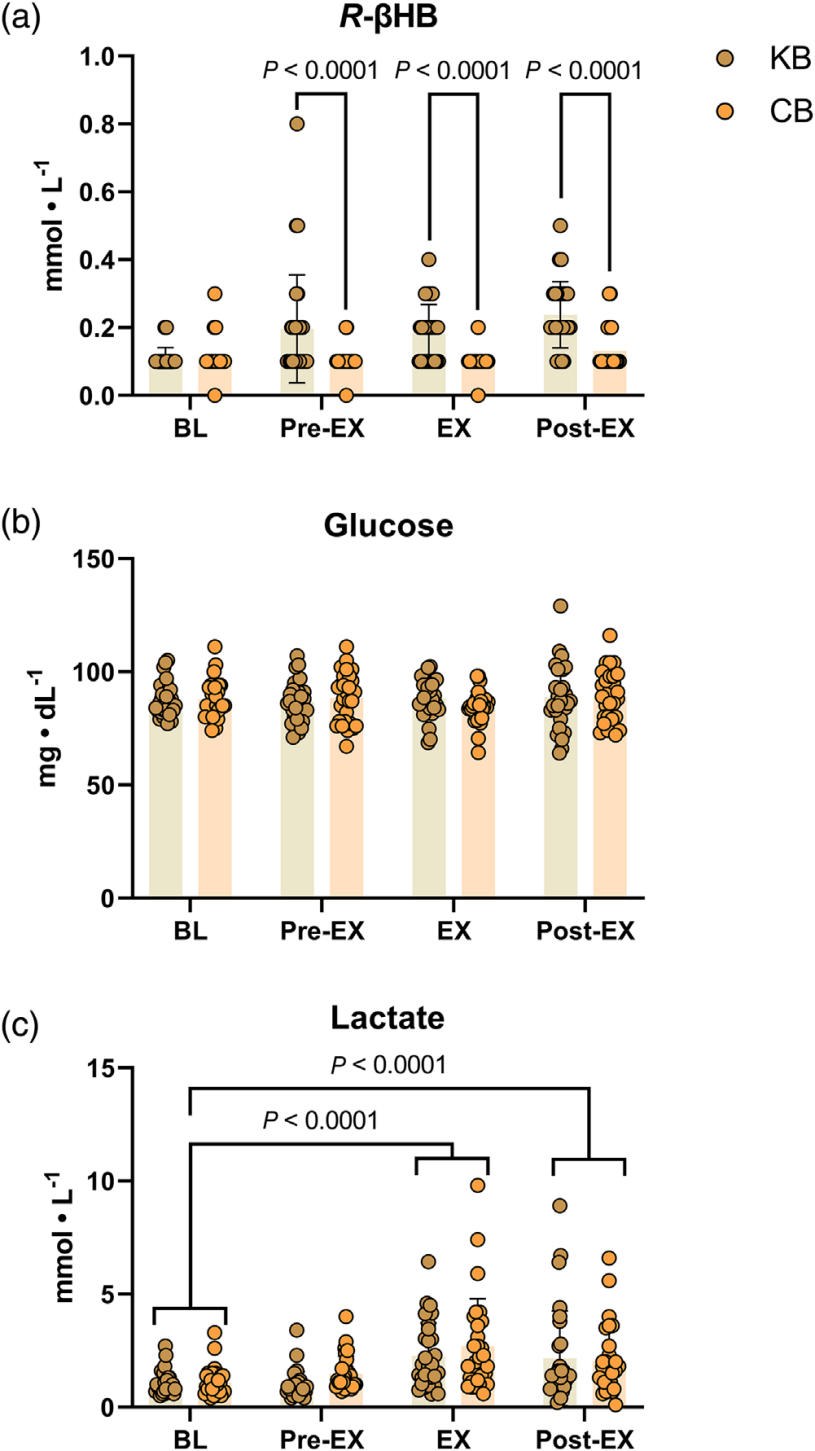
Capillary blood results. Capillary ketones (*R*‐βHB) (a), glucose (b), and lactate (c) were measured enzymatically in whole blood at baseline (BL), 3 h after KB/CB ingestion and pre‐exercise (Pre‐EX), during exercise (EX) and immediately post‐exercise (Post‐EX). There were no significant differences between‐conditions at BL. A 2 (condition) × 4 (time) repeated measures ANOVA model revealed significant within‐time, between‐condition, and interaction effects for *R*‐βHB (all *P* < 0.0001). There were no significant changes in glucose. Lactate increased significantly over time at EX and Post‐EX relative to BL (*P* < 0.0001), independent of the condition. Data presented as means ± SD. CB, carbohydrate bars; KB, ketogenic bar.

There were no differences between participant metabolite concentrations while fasted and rested at baseline (Table 6). There were significant *R*‐βHB changes detected within‐KB, between conditions, and significant interaction effects (all *P* < 0.0001; Figure 4a). Capillary *R*‐βHB concentrations increased marginally from baseline to pre‐exercise (∆: 0.1 ± 0.02 mmol/l; *P* < 0.0001), an effect that was sustained thereafter throughout post‐exercise measurements (mean *R*‐βHB: ∼0.2 mmol/l; *P* < 0.0001). There were no significant *R*‐βHB effects in the CB condition.

**TABLE 6:** Blood metabolites (*n* = 29).

		Time point				
Variable	Condition	BL	Pre‐EX	EX	Post‐EX	2 × 4 RM‐ANOVA
*R*‐βHB (mmol/l)	KB CB	0.11 ± 0.03 0.11 ± 0.05	0.20 ± 0.16 0.10 ± 0.03	0.18 ± 0.07 0.10 ± 0.02	0.24 ± 0.09 0.13 ± 0.06	Time, *P* < 0.0001 Condition, *P* < 0.0001 Interaction, *P* < 0.0001
Glucose (mg/dl)	KB CB	87.7 ± 7.6 87.8 ± 8.3	86.7 ± 9.1 88.4 ± 10.9	88.3 ± 8.4 84.3 ± 7.0	88.7 ± 14.3 89.0 ± 11.3	Time, *P* = 0.467 Condition, *P =* 0.635 Interaction, *P =* 0.209
Lactate (mmol/l)	KB CB	1.08 ± 0.52 1.11 ± 0.66	0.98 ± 0.64 1.48 ± 0.79	2.34 ± 1.48 2.84 ± 2.10	2.26 ± 2.14 2.09 ± 1.53	Time, *P* < 0.0001 Condition, *P* = 0.085 Interaction, *P* = 0.199

Data shown as means ± SD. Finger capillary blood glucose, *R*‐β‐hydroxybutyrate and lactate were assessed across four time points. The data was collected at 10‐min intervals during EX and are presented as the grand mean. Abbreviations: BL, baseline; CB, carbohydrate bars; EX, exercise; KB, ketogenic bar; RM, repeated measures.

Neither the KB nor CB influenced glycaemia during the study. Participants demonstrated normal glycaemia at baseline (glucose < 100 mg/dl) (Figure 4b). Capillary glucose values remained generally stable throughout the KB and CB conditions (88 ± 0.9 vs. 87 ± 2.1 mg/dl; *P* = 0.635), recording a ∼2% coefficient of variation throughout all four time point measurements.

Lactate concentrations did not change significantly from the baseline resting phase to pre‐exercise. Average lactate values increased significantly during exercise relative to baseline, independent of the condition (∆: 1.4 ± 1.7 mmol/l; *P* < 0.0001), an effect that was sustained beyond baseline at the post‐exercise measurement (Figure 4c).

### 3.4 Perceptual responses

**FIGURE 5:**
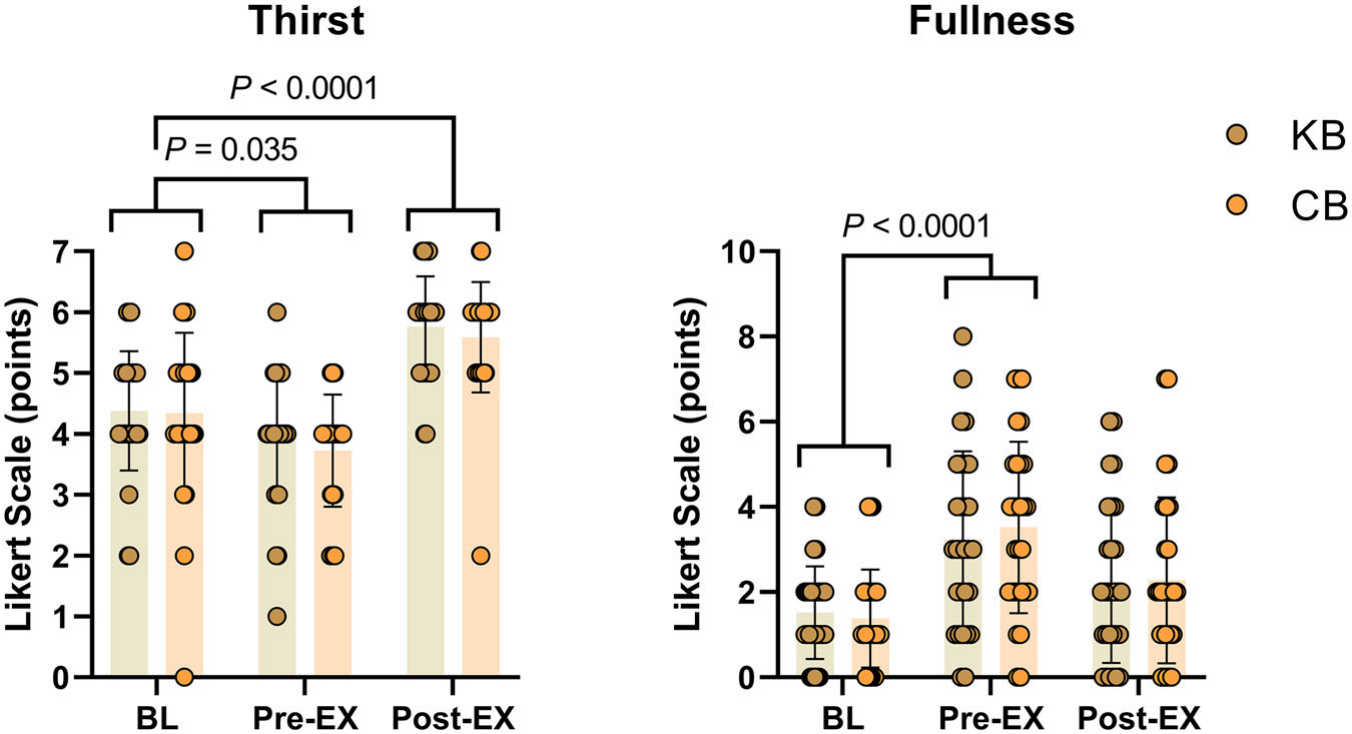
Perceptual results. Subjective measurements of thirst and fullness were assessed via validated Likert‐scale surveys. Participants completed three surveys: before consuming the KB/CB at baseline (BL), 3 h later and pre‐exercise (Pre‐EX), and immediately post‐exercise (Post‐EX). There were no differences in either thirst or fullness between conditions at BL. A 2 (condition) × 3 (time) repeated measures ANOVA revealed significant time effects, but no condition or interaction effects. Thirst measures decreased significantly from BL to Pre‐EX (*P* < 0.05), whereas Post‐EX thirst increased significantly relative to BL (*P* < 0.001). Fullness measures increased significantly from BL to Pre‐EX (*P* < 0.001) with a return to BL values Post‐EX. Data presented as means ± SD. CB, carbohydrate bars; KB, ketogenic bar.

There were no significant differences between participant subjective thirst and fullness measures at baseline (Table 7). Surveys for both perceptual parameters demonstrated a significant main effect of time (*P* < 0.001), but no between‐condition or interaction effects. Subjective measures of thirst decreased significantly from baseline to pre‐exercise (∆: −0.5 ± 1.5; *P* < 0.05), followed by a significant increase immediately post‐exercise (∆: 1.3 ± 1.3; *P* < 0.001). Subjective measures of fullness increased from baseline to pre‐exercise (∆: 1.9 ± 2.3; *P* < 0.001), an effect that returned to baseline values post‐exercise (Figure 5).

**TABLE 7:** Thirst and fullness measures.

		Time point			
Variable	Condition	BL	Pre‐EX	Post‐EX	2 × 3 RM‐ANOVA
Thirst	KB CB	4.4 ± 0.9 4.3 ± 1.3	3.9 ± 1.0 3.7 ± 0.9	5.8 ± 0.8 5.6 ± 0.9	Time, *P <* 0.001 Condition, *P =* 0.396 Interaction, *P =* 0.753
Fullness	KB CB	1.5 ± 1.1 1.4 ± 1.1	3.2 ± 2.1 3.5 ± 2.0	2.2 ± 1.8 2.3 ± 1.9	Time, *P <* 0.001 Condition, *P* = 0.687 Interaction, *P* = 0.546

Data shown as means ± SD. Thirst scale (1: ‘not thirsty at all’, 7: ‘very, very thirsty’); Fullness Scale (0: ‘empty’, 10: ‘extremely full’). All participants received 500 ml of water at BL to control for thirst confounders. BL, baseline; CB, carbohydrate bars; EX, exercise; KB, ketogenic bar.

## 4 DISCUSSION

### 4.1 Key findings

Twenty‐nine, recreationally active and military phenotype males were able to sustain ∼1 h of steady‐state incline rucking under 30% of body weight load. Consuming isocaloric KB or CB 3 h before the ruck did not differentially modulate TTE. Athletes demonstrated good metabolic flexibility during submaximal workload as revealed by increased lipid or carbohydrate oxidation rates after KB or CB ingestion, respectively. Capillary *R*‐βHB was augmented by the KB, likely attributed to the medium‐chain triglycerides in the KB formulation and lower ${\dot{V}}_{CO_{2}}$. Glycaemia was unaffected by condition. Exercise increased lactate predictably during exercise and post‐exercise, and trended higher in the CB condition relative to KB. Perceptual measures of thirst and fullness were similar between conditions.

### 4.2 Performance was comparable between the KB and CB

Participants spent on average 1 h rucking, or approximately 3 km, before reaching volitional exhaustion. Previous ruck studies have implemented longer ruck designs, flatter terrain, heavier rucks, non‐steady‐state components (i.e., running, variable temperature, uneven terrain), or no pre‐event nutrition (Army, 2022; Looney et al., 2022; Walsh et al., 2020), and therefore our simulation must be viewed with the understanding of precise laboratory conditions.

When accounting for all the physiological parameters and ruck grade, predictive ruck metabolic equations revealed that the average participant in this study expended 270 kcal/h (Looney et al., 2022). Our original speculation was that rucking would induce an energy expenditure rate closer to 1000 kcal/h (Jacobs et al., 1989; Tassone & Baker, 2017; Walsh et al., 2020) and create an energy gap that would be filled by the nutritional bars (Hirsch et al., 2005; McClung et al., 2020). Although we observed significantly less energy expenditure during our simulated ruck, the nutritional KB and CB bars consumed pre‐ruck were non‐limiting to performance. Rucking over longer distances, with a lower percentage grade, greater speed and modifiable environmental temperature should be considered in the future for improving the ecological validity of modern military exercises.

### 4.3 Macronutrient ingestion determined substrate oxidation preference

The RER and substrate oxidation rates during exercise corroborated the main macronutrient ingested 3 h before the ruck. Mean relative oxygen consumption in our study (∼53% of ${\dot{V}}_{O_{2}\max}$) was maintained between conditions; therefore, the KB to CB comparisons were interpreted within the context of similar ruck effort. While participants did not attain the ‘fat‐max’ range that we originally expected (Achten & Jeukendrup, 2003; Lambert et al., 1997), the pre‐determined speed and grade for this ruck were adequate for eliciting intensities often expected during military operations (Army, 2022). Acute KB ingestion increased fat oxidation during the ruck beyond the CB (Δ: ∼0.2 g/min), whereas the CB increased mean carbohydrate oxidation relative to the KB (Δ: ∼0.6 g/min). In other words, for every gram of lipid oxidized during the KB condition, 3 g of carbohydrate was oxidized during the CB condition to sustain equal ruck workload.

### 4.4 Blood analytes

Capillary *R*‐βHB was elevated hours after the KB ingestion, revealing that the medium‐chain triglycerides within the KB matrix predictably influenced ketonaemia (Décombaz et al., 1983). Whereas *R*‐βHB effects (>0.5 mmol/l) have been previously tested for athletic performance using a ketogenic diet or acute ingestion of exogenous ketone supplements (Cox et al., 2016; Evans et al., 2022; Kackley et al., 2020; LaFountain et al., 2019), this investigation modified pre‐exercise macronutrient ingestion rather than providing a robust ketone supplement per se. The capillary *R*‐βHB results must be viewed with the understanding that there are (1) unclear effects in the literature attributing power, work capacity, or speed to *R*‐βHB directly (Evans et al., 2022; Valenzuela et al., 2020); and (2) the magnitude *R*‐βHB change that we detected in this study was unlikely to have influenced ruck performance. The nutritional bars also did not exert any adverse effects on capillary glucose – despite large differences in total carbohydrates (Δ: 148 g) and sugar (Δ: 68 g) – although predictable given the 3 h gap between pre‐ and post‐ingestion measurements. Moreover, lactate was also unaffected by condition and increased predictably with ruck exertion independent of the bar consumed. These results collectively suggest that healthy, recreationally active athletes may consume either a lipid‐ or carbohydrate‐dense nutritional bar before rucking without experiencing detrimental blood responses, thereby demonstrating elements of individualized nutrition (Karl et al., 2022).

### 4.5 Perceptions of thirst and fullness

Self‐reported thirst perceptions were replicated between experimental visits. Each participant consumed a fixed amount of water (500 ml) before consuming the nutritional bar to minimize confounding responses. Both the KB and CB provided sodium equivalent of one‐third of daily sodium requirements for US adults (mean: 835 mg; ∼2 g of table salt), and decreased thirst perception from rest to 3 h post‐ingestion. This effect was surprising because sodium was expected to increase subjective thirst; however, it only increased after exercise as confirmed by the surveys, and is likely attributed to sweat loses during the ruck (Stachenfeld, 2008). Moreover, we predicted that the CB volume relative to the KB (Δ: 32%) and carbohydrate content, specifically fibre content (Δ: 12 g), should have increased fullness measures beyond the KB; however, this was not the case in our investigation. Second, we expected that fat and carbohydrate would differentially modulate fullness sensations through humoral signalling conferred by unique carbohydrate and fat digestion (Karhunen et al., 2008). Nevertheless, this was also not the case, as fullness increased significantly before exercise and independent of the bar consumed, followed promptly by a return to baseline values after exercise (∼4 h after ingestion). Measurements of circulating blood hormones, such as leptin, ghrelin and other hormones/peptides involved in fullness and satiety mechanisms (Christ et al., 2006), may provide greater insight into how these subjective perceptions are modulated by pre‐ruck nutrition.

### 4.6 Limitations and future directions

This novel pre‐ruck nutritional investigation produced many observations that have not been realized in other studies. Despite these observations, there were several limitations that we feel should be addressed. While a healthy population was used in this study, we did not assess active‐duty service members, which could have led to differences in performance outcomes. The exercise protocol used was like military rucking events; however, lack of rucking experience, including unfamiliarity with the load of the ruck, could have skewed the results in the studied population. Using a population of active‐duty service men and women and who actively participate in rucking activities should be explored in the future to address military‐specific outcomes.

A methodological strength was standardizing all participants to consume the Standard American Diet during and between experimental periods to control for diet confounders (Shan et al., 2019). We are aware that military camp nutrition may not resemble this dietary strategy. Therefore, more work may be required to verify ecological validity of our snacking alternatives in the field. A closer examination into how the KB and CB can complement field rations, not replace them, may be prudent to focus on, as this information can advance military research on field nutrition alternatives. Additionally, future research should focus on assessing similar pre‐exercise fuel ingestion using distances and time standards relevant to a military setting, plus soldier‐specific performance parameters, including measures like cognitive function and marksmanship, to increase military performance relevance.

### 4.7 Conclusion

Energy‐dense nutritional bars, containing complex carbohydrates or lipids, sustained ∼1 h of steady‐state, graded rucking with a 30% of bodyweight rucksack. Athletes demonstrated good metabolic flexibility as reflected by the primary substrate oxidation shift during exercise. The KB elicited a modest increase in capillary ketones, whereas glycaemia remained stable in both conditions. While lactate increased predictably with exercise, more work is needed to evaluate how acute carbohydrate or lipid ingestion before exercise modulates lactate appearance during submaximal effort. Both bars were equally as filling and did not stimulate thirst differently. The weight and energy density advantage conferred by a portable and lipid‐rich, pre‐event nutritional bar merits further evaluation.

## AUTHOR CONTRIBUTIONS

Philip J. Prins, Andrew P. Koutnik and Dominic P. D'Agostino conceived the original study design. Philip J. Prins, Jeffrey D. Buxton, Alex Buga, Shawn Cline, Trevor Fleck, Jared Krout and Doran Newby conducted participant testing and collected all the data. Philip J. Prins, Alex Buga, Justen T. Stoner, Drew D. Decker and Bradley T. Robinson conducted data analysis. Philip J. Prins, Alex Buga, Chris D. Crabtree, Justen T. Stoner, Drew D. Decker, Bradley T. Robinson, Madison L. Kackley and Teryn N. Sapper drafted the first manuscript. Philip J. Prins, Alex Buga, Andrew P. Koutnik and Jeff S. Volek drafted the final manuscript. All authors have read and approved the final version of this manuscript and agree to be accountable for all aspects of the work in ensuring that questions related to the accuracy or integrity of any part of the work are appropriately investigated and resolved. All persons designated as authors qualify for authorship, and all those who qualify for authorship are listed.

## CONFLICT OF INTEREST

Andrew P. Koutnik is a patent inventor (US11452704B2) and has consulted for Simply Good Foods. Dominic P. D'Agostino is the owner of Ketone Technologies LLC, which does research consulting and public speaking events. Jeff S. Volek received royalties for books on low‐carbohydrate diets; is founder and has equity in Virta Health; and serves on the advisory board of Simply Good Foods.

## Supporting information

Statistical Summary Document

## Data Availability

The raw data that was analysed for this manuscript will be made available and provided upon reasonable request.
